# A problem of gendered injustice? Objective and subjective poverty among older women and men across European welfare regimes

**DOI:** 10.1007/s10433-023-00796-5

**Published:** 2024-01-03

**Authors:** Camilla Härtull, Mikael Nygård

**Affiliations:** Åbo Akademi University, PB 311, 65101 Vaasa, Finland

**Keywords:** Objective and subjective poverty, Old age, Gender, Europe, Welfare regimes, European Social Survey

## Abstract

Using European Social Survey data, this article studies the prevalence of objective and subjective poverty among older women and men (60+ years) in 21 European countries. Objective poverty refers to whether one’s disposable income falls below the poverty line, whereas subjective poverty relates to the capacity to make ends meet. It analyzes gender differences in these two dimensions of poverty and the role of gender as an explanation to these phenomena while controlling for other individual-level variables as well as the role of welfare state regimes. The results show that older women are more exposed to objective poverty than men, and that female gender remains strongly and positively correlated with this kind of poverty even when controlling for other variables. They also show that other individual-level variables, such as partnership, paid work and education curbs objective poverty, while the type of welfare regime does not matter. As to subjective poverty, on the other hand, there is no significant association with female gender, nor with the type of welfare regime, while individual-level variables such as subjective health, partnership and paid work are negatively correlated with this dimension of poverty. Subjective poverty is somewhat more influenced by contextual factors than objective poverty although the type of welfare state regime is not significantly associated with subjective or objective poverty.

## Introduction

During the post-WWII era, income inequality between older women and men in Europe has diminished due to higher labor participation of women and the expansion of pension rights (e.g., Arza and Kohli 2008). However, according to the European Commission (2021) as well as the OECD (2019), Europe still suffers from old-age poverty risks disfavoring women. The observed gender gap in old-age poverty risks amounts to four percentage points in the ages above sixty-five for the European union as a whole (European Commission 2021). This problem concerns primarily rudimentary welfare states (e.g., Haan et al. 2017), but is also prevalent in the Nordic countries known for their high levels of gender equality and extensive pension systems (Härtull and Nygård 2014; Nygård et al. 2017; Riekhoff and Järnefelt 2017).

Gendered poverty is not a new phenomenon. It has been suggested that this problem is related to the overall weaker labor market position of women, their lower wage levels, their larger responsibilities for domestic unpaid work and the pensions they receive when retiring (Gough 2001; Nygård et al. 2017; Tuominen et al. 2011). Gendered poverty in old age thus represents one important facet of injustice in today’s welfare states and needs to be given high priority, for instance within pension politics. This statement is underlined by the fact that the world’s population is aging and that the older population will predominately be female, since women on average live longer than men (UNDESA 2017). Yet the literature on old-age poverty is still limited, and even less focus has been put on gender differences in this respect (Kabeer 2015; Kwan and Walsh 2018). The existing research in this area consists of mainly national studies (e.g., Riekhoff and Järnefelt 2017), with only occasional comparative studies (e.g., Möhring 2015) focusing on the role of institutional factors for gender differences in old-age poverty.

Therefore, the aim of this article is to study the prevalence of objective and subjective poverty among older adults (60+ years) in 21 European countries and to test whether this prevalence can be explained mainly by gender when simultaneously controlling for other individual-level variables as well as contextual factors residing on the country level. Objective poverty refers to whether one’s disposable income falls below a given poverty line, whereas subjective poverty relates to the capacity to make ends meet. The overall aim can be divided into three main research questions. First, the article examines the prevalence of objective and subjective poverty among older women and men in different countries. Second, it tests whether this prevalence is explained by gender while simultaneously controlling for other individual-level variables, such as educational level or civil status. Third, it assesses how much of the variance in objective and subjective poverty resides on a country level and what the explanative power of different welfare state regimes is. To this end, we use survey data from the 2016 European Social Survey that allows a multidimensional and multilevel approach to poverty analysis. The article contributes not only to the scarce literature on gender differences in old-age poverty, but also adds to the multidimensional analysis of poverty. Furthermore, it studies the importance of context by evaluating how much of the gender differences in poverty resides on a country level and what role welfare state regimes play for gendered old-age poverty.

The rest of the article is structured as follows. The next section presents a review of previous research in this field, the third section the data and methods used and the fourth section the findings. In the final section, the results are summarized and discussed.

## Literature review

Old age is related to higher risks of poverty, although the emergence of modern welfare states has mitigated these risks to some extent (Thelin 2013; Scharf and Keating 2012). Moreover, poverty in old age is also greatly a gendered problem, since it is mainly women who face income poverty when they get old (Nygård et al. 2017; Riekhoff and Järnefelt 2017; Kabeer 2015; Ahonen and Bach-Othman 2010; Shaw and Lee 2005; Smeeding and Sandström 2005; Lister 2004; Bianchi 1999). This problem of social injustice is not new—the concept of ‘feminization of poverty’ was introduced by Diana Pearce (1978) already in the 1970s. And even if gender differences in old-age poverty have received less attention than working-age poverty (Ahonen and Bach-Othman 2010), there is growing awareness of this problem. For example, Haan et al. (2017) show that in Germany, poverty rates among pensioners are expected to rise in the coming decades, and that single women are at a particular risk. This problem has been highlighted also in Nordic countries (e.g., Government of Sweden 2013; Kuivalainen et al. 2017; Nelson et al. 2019). In Finland and Sweden, for example, the systematically higher levels of average pensions for men have frequently been up to debate (e.g., Kuitto and Kuivalainen 2021; Ahonen and Bach-Othman 2010; Flood 2014; Sjögren Lindquist and Wadensjö 2012).

The explanations of gender differences in old-age income poverty vary across scholars. Some observers have considered poverty among women in developed welfare states to be a structural disadvantage inherent in the contemporary welfare states (e.g., Walker and Foster 2014). Not only do women experience more uneven working careers and greater domestic responsibilities than men, but they also often face another structural inequality in terms of lower pensions generated by income-related pension schemes (Arber 2006; Price and Ginn 2003). If we look at female labor participation on a general level during the period between 1970 and 1990, women in Western democracies acquired roughly the same level of education as men, implying that the gender wage gap cannot be accounted for by differences in human capital (Arber 2006; Lister 2004). Instead, we need to look for answers in the fact that women generally have chosen employment in different sectors of the labor market than men, tended to stay employed in the same position for a longer time than men, and often had their careers disrupted by childbirths and domestic responsibilities (Gough 2001; Middleton 2002; Orloff 2009). In addition, the average retirement age of women is almost two years lower than that of men in most OECD countries (OECD 2019).

Together, these circumstances account for a large share of the wage gaps, and consequently also for the differences in old-age poverty risks. The vulnerable economic situation of older women who live alone has also been emphasized. The poverty risk for this group of women is a fact in several developed countries such as Belgium (Peeters and de Tavernier 2015), Germany (Haan et al. 2017) and United Kingdom (Gornick et al. 2009).

The impact of household size has also been recognized in one of the few existing comparative studies on gender differences in old-age poverty (Ahonen and Bach-Othman 2010). It shows that gender differences in income poverty are higher in EU-countries where women participate very actively in paid labor. This may seem somewhat surprising, since a higher degree of labor market participation during one’s active life could be expected to lower the poverty risk in old age (Kautto 2011). In contrast, Ahonen and Bach-Othman (2010) found that size and type of household is somewhat stronger linked to the risk of facing income poverty in old age. In countries where older women often live in large households, such as Southern European countries, the gender differences in old-age income poverty risks are less accentuated than in countries where older females more commonly live alone.

In another comparative study comprising thirteen European countries, Möhring (2015) examined how national pension systems shape the relationship between one’s previous employment history and the individual pension income. For men, only little cross-national variation was found, while for women, the relationship varied across countries.

Moreover, Möhring (2015) found that atypical employment histories do not automatically go along with low income in old age, because the design of the pension system plays a key role in compensating for gender inequalities of the working life. Still, inequalities between men’s and women’s old-age income rather remain related to the gendered division of market and care work than to pension schemes. As an example, Möhring (2015) states that the negative effect of mother’s atypical employment patterns on the pension income remains even after controlling for country differences. Since women choose part-time jobs to be able to combine work with care responsibilities, “working mothers face a ‘pension penalty’ irrespective of the specific national institutional context” (Möhring 2015, 21).

This result relates to the fact that, over time, there has been a gradual convergence of European welfare regimes as well as their pension systems (Hemerijck 2013), although there is considerable variation in how such systems function and what their redistributive capacities are. In the early-1900s, Continental and Easter European countries laid the foundations for relatively generous, income-related and insurance-based pension systems sustaining a male breadwinner model. After WWII, many Eastern European countries under communism changed these systems to support a dual-earner model (Ebbinghaus 2011). In the Nordic, as well as the Anglo-Saxon countries and the Netherlands, there was a stronger accentuation of universal and citizen-based social rights, which also tax-funded basic pension (ibid.). However, over time, these countries also introduced insurance-based and income-related pension schemes creating so called ‘two-pillar’ systems that in combination with a higher female labor market participation and a stronger emphasis of a dual-earner model led to higher degree of gender-related equality than in Continental and Southern Europe (Möhring 2015). Since the 1990s, however, also many of the Continental and Southern European countries have taken steps toward a dual-earner model and ‘two-pillar’ pension systems. For instance, both in Spain and Germany tax-funded basic pension systems were introduced in the 1990s or early-2000s as a way of combatting poverty among older persons, and notably older females (Hemerijck 2013; Arza and Kohli 2008). Moreover, most European countries have introduced privately funded occupational or voluntary pensions creating ‘three-pillar’ systems (Ebbinghaus 2019).

Nordic countries stand out in terms of low degrees of income inequality and poverty. However, although the poverty rate among older Scandinavians plunged drastically after the 1960s due to the introduction of modern pension schemes (Fritzell and Ritakallio 2010; Gustafsson et al. 2009), older females still face a higher poverty risk than men in countries like Finland and Sweden (Kuitto and Kuivalainen 2021; Arber 2006; Ebbinghaus 2019; Nygård et al. 2017; Price and Ginn 2003; Zaidi 2010).

Because of the complexity of poverty as a social phenomenon, various measures have been developed to capture this problem. Commonly used measures set the poverty line at a certain percent of the national median income, or at a certain level of income, beneath which basic needs of life cannot be met (European Anti-Poverty Network 2014). However, measures based on a national level aggravates country comparisons if the gap in living standards between the richest and poorest countries is large. Furthermore, objective income measures have been criticized because they do not necessary reflect what individuals themselves consider as social necessities (Duvoux and Papuchon 2019; Fahey 2007). Subjective approaches to poverty, on the other hand, give an assessment of the practical impacts that a lack of resources may have (Alcock 2006; Ravallion 2012). Subjective comparisons of one’s situation with others may however bias the results as individuals tend to be satisfied with their situation when their incomes increase but less satisfied when other people’s incomes increase (Clark 2018). There is also a conceptual distinction between objective and subjective assessments of poverty in that the former is about low incomes, whereas subjective poverty is an individual’s judgment that gives more weight to wealth, which helps to make ends meet when incomes are low (Marks 2007). Persons in old age may have low incomes but a greater amount of wealth, such as savings, which are of primary importance in stabilizing consumption and may be at least as important as income in explaining subjective well-being (Brulé and Suter 2019; Headey et al. 2008).

Nevertheless, a subjective indicator of poverty brings an added value because the question on whether one feel that it is easy or difficult to get by with the present income captures economic stress and allows to examine the extent to which welfare states are able to decrease economic vulnerability (Baldini et al. 2018).

In this regard, age can be said to have a twofold effect on poverty. On the one hand, aging in itself may influence (pension) incomes and the amount of accumulated wealth, but on the other hand, there may also be an age cohort effect that relates to when a person is born and what kind of labor market prospects and pension rights that person faces.

To conclude so far, previous research suggests that old-age poverty is strongly related to a person’s gender. The use of subjective measures alongside objective ones is of utmost importance also when focusing on elderly, given that previous research point to notable variation across sexes in the subjective experiences of poverty (Thelin 2013). Moreover, there is a growing literature stressing the merits of using multiple indicators, particularly in light of the UN Sustainable Development Goals. The need for a multidimensional approach to poverty has resulted in several measures such as the Human Development Index (HDI) and the Global Multidimensional Poverty Index (MPI). As our data do not allow using any of these, our multidimensional approach to is to use two different measures as described in the next section.

## Data, variables and methods

### Data

We used data from the 2016 round of the European Social Survey.^1^ This cross-national survey is conducted every second year as face-to-face interviews and it measures attitudes, beliefs and behavior patterns in more than thirty countries. (European Social Survey 2019). The 2016 wave comprises 23 countries, Austria, Belgium, Czech Republic, Germany, Estonia, Finland, France, Great Britain, Hungary, Iceland, Ireland, Italy, Israel, Lithuania, Netherlands, Norway, Poland, Portugal, Russian Federation, Slovenia, Spain, Sweden and Switzerland. However, we chose to omit Israel and Russian Federation, since we wanted to focus only on European countries. When discussing old age, it is important to remember that any age categorization entails a certain amount of arbitrariness, and that old age has different meanings in different cultures and times (Wilson 2000). Following the definition used by UNDESA (2017), we restrict the data to persons aged sixty and older. Respondents with missing information on any of the two outcome variables are excluded from the study. In total, we analyze 10,960 respondents, whereof 45.7% are men and 54.3% women (Table 1).

**Table 1:** Distribution of control variables

N = 10,960	%	Distribution by gender (%)	
Country		Men	Women
Austria	4.2	4.1	4.3
Belgium	5.3	5.7	4.9
Switzerland	3.6	3.9	3.4
Czechia	5.2	5.0	5.4
Germany	8.3	8.4	8.1
Estonia	6.3	5.3	7.2
Spain	4.5	4.7	4.4
Finland	6.2	6.2	6.2
France	6.0	6.0	6.1
United Kingdom	4.5	4.8	4.2
Hungary	3.6	3.1	4.0
Ireland	5.0	5.5	4.6
Iceland	1.9	2.1	1.6
Italy	5.2	5.1	5.2
Lithuania	5.5	4.4	6.4
Netherlands	4.6	5.0	4.4
Norway	4.1	4.7	3.6
Poland	4.2	3.8	4.5
Portugal	3.5	3.7	3.4
Sweden	4.7	5.0	4.4
Slovenia	3.5	3.5	3.6
Total	100.0	100.0	100.0
Gender			
Men	45.7		
Women	54.3		
Total	100.0		
Age			
60–69	53.2	54.2	52.4
70 or older	46.8	45.8	47.6
Total	100.0	100.0	100.0
Lives with partner			
Yes	65.2	77.8	54.7
No	34.8	22.2	45.3
Total	100.0	100.0	100.0
Domicile			
Urban	57.7	56.9	58.3
Rural	42.3	43.1	41.7
Total	100.0	100.0	100.0
Subjective general health			
Good	45.1	49.3	41.6
Fair	41.2	38.9	43.1
Poor	13.7	11.8	15.3
Total	100.0	100.0	100.0
Educational level			
Low	41.2	36.8	45.0
Intermediate	42.9	44.9	41.2
High	15.9	18.3	13.8
Total	100.0	100.0	100.0
In paid work			
Yes	17.3	20.3	14.7
No	82.7	79.7	85.3
Total	100.0	100.0	100.0
Regime			
Continental	32.0	33.0	31.1
Southern	13.2	13.5	13.1
Anglo	9.6	10.4	8.9
Eastern	28.3	25.1	31.1
Nordic	16.9	18.0	15.8
Total	100.0	100.0	100.0

Percentages are calculated on weighted data (pweight and pspwght)

### Variables

**Fig. 1:**
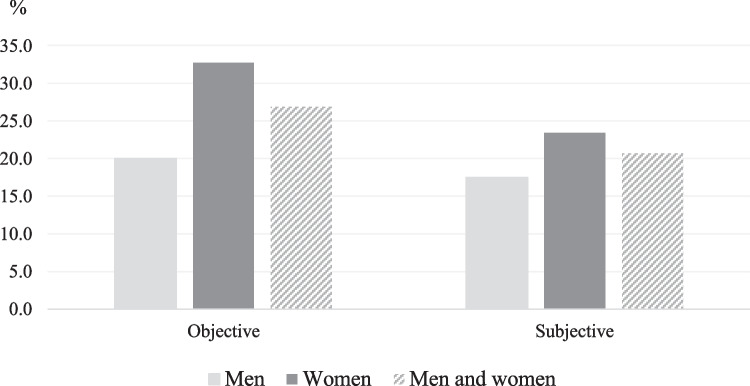
Objective and subjective poverty (%) in older adults in 21 European welfare regimes by gender

We use two outcome variables for measuring poverty in a household context (cf. Ravallion 2012): one representing objective and the other subjective poverty. *Objective poverty* is based on the respondent’s estimation of which income decile her/his household’s net income would fit in. Each of the ten income categories broadly corresponds to deciles of the actual household income range in all respondents in each country. We categorize respondents in our study sample as poor if they are found in the two lowest deciles, that is, the lowest quintile. Since the deciles are based on the entire sample and a relatively higher share of old-age persons are found in the lowest deciles, the overall objective rate exceeds 20% (Fig. 1). Our objective poverty indicator is constrained by the data used, but defining the lowest quintile as poor is a strategy previously used by Atkinson et al. (1999). *Subjective poverty* relates to whether a respondent feels that it is easy or difficult to get by with the household’s present net income. Respondents feeling that it is difficult or very difficult to get by, are categorized as poor, while non-poor relate to those who report that they are coping or living comfortably on their income. In earlier research on subjective poverty, a similar measure has been used by Posel and Rogan (2014). This process is of course not straightforward and can be discussed. For example, it could be argued that those feeling difficult on present income could have been classified as subjectively non-poor, since most people probably face some kind of economic difficulties at some point during their lives. However, as been pointed out in discussions on measuring poverty (c.f. Chen and Corak 2008), the process inevitably involve value judgments, and the chosen poverty line will indeed affect the poverty rates, whether it be a question of objective or subjective poverty.

The main explanatory variable is *gender*. We also use a set of individual-level control variables that have been shown to influence poverty and that were dictated by previous research on older persons’ incomes or poverty (e.g., Ahonen and Bach-Othman 2010; Kwan and Walsh 2018). These variables are *age*, whether the respondent *lives with a partner*, type of *domicile* (rural or urban), *self-rated health* (poor, fair or good), *educational level* (low, intermediate or high), and whether the respondent is currently in *paid work*. The variable age separates respondents who are aged sixty to sixty-nine from those who are seventy or older. Type of domicile is dichotomized so that the respondents living in big or small cities or in suburbs of big cities are categorised as urban, while rural inhabitants are represented by those living in a country-village or in the countryside. Self-rated health is categorized into good (respondents with very good or good health), fair (respondents with fair health) and poor (respondents with bad or very bad health). Based on the respondent’s highest level of education, low, intermediate and high education refers to those with ISCED I-II, ISCED III-IV and ISCED V (or higher) level education, respectively.

The overall distribution of outcome variables and control variables is found in Table 1 alongside the distribution of these variables by gender. As can be seen from the table, a higher proportion of men than women lives with a partner, reports good general health, has a higher education and is still in paid work, while living in an urban area is somewhat more common in women.

On the context level, we used dummy variables capturing the variance in welfare-institutional characteristics, including the variance in pension rights. Based on previous work (e.g., Hemerijck 2013), we categorized Austria, Belgium, Switzerland, Germany, France and the Netherlands as *Continental*, Czech Republic, Estonia, Hungary, Lithuania, Poland and Slovenia as *Eastern European*, Spain, Italy and Portugal as *Southern European*, the United Kingdom and Ireland as *Anglo-Saxon*, and Finland, Iceland, Norway and Sweden as *Nordic* welfare state regimes. While being crude and somewhat problematic (e.g., in the case of the Netherlands, which is often considered as a hybrid, Social-democratic/Continental welfare regime), this analytical approach still enable us to indirectly discriminate the role of welfare state regimes for the prevalence of old-age poverty.

### Analyses

In the first stage of the analysis, the distribution of the two outcome variables, objective and subjective poverty, was assessed per country, gender and other background variables in order to gain an overall pattern of the prevalence of the two aspects of poverty among older females and males (Table 2). The second step was to use multilevel regression models for assessing the role that gender has for the prevalence of these two indicators of income poverty when simultaneously controlling for the individual-level and contextual-level control variables. In this step, we also calculated the share of the variation in poverty residing on the country level (Table 3 and 4). We used the *xtmelogit* command in Stata 17. In the analysis, post-stratification and population size weights were applied in accordance with recommendations by ESS.^2^ We fitted five logistic regression models for each outcome variable, the first model (M0) providing a baseline estimate of the level-2 variance (σ2u) as well as the variance partition coefficients (VPC) in accordance with Snijders and Bosker’s (2001) formula. The next model (M1) assesses the effect of gender while simultaneously controlling for age, partnership and residence, and the subsequent model (M2) adds the effects of health and education. Model 3 (M3) provides the estimates for the four dummy variables assessing welfare-institutional configurations (using the Nordic model as reference). The final model (M4) is a full model including estimates for all variables.

**Table 2:** Share of older adults experiencing objective and subjective poverty, by gender and background variables

		Objective poverty (%)		Subjective poverty (%)	
		Men	Women	Men	Women
Country	Austria	19.1	24.9	9.6	11.7
	Belgium	16.1	26.1	19.6	21.4
	Switzerland	21.4	31.3	9.5	10.8
	Czechia	18.3	41.3	37.9	47.9
	Germany	16.6	24.9	6.5	9.2
	Estonia	24.0	41.1	29.3	34.9
	Spain	29.6	43.6	24.5	30.0
	Finland	17.6	27.6	8.5	10.2
	France	15.8	29.0	10.4	13.4
	United Kingdom	26.5	30.9	6.2	5.9
	Hungary	28.7	48.8	33.1	45.2
	Ireland	30.0	33.6	12.5	15.8
	Iceland	20.4	21.4	6.5	9.4
	Italy	20.1	32.4	30.0	40.8
	Lithuania	21.5	40.3	44.0	50.8
	Netherlands	9.2	15.9	3.2	7.5
	Norway	16.5	28.0	2.9	4.3
	Poland	20.2	37.9	31.7	32.2
	Portugal	21.9	36.0	33.8	48.3
	Sweden	12.9	30.6	7.8	7.0
	Slovenia	22.7	35.7	16.3	18.3
Age	60–69	17.6	24.9	18.0	23.2
	70 or older	22.9	41.3	16.9	23.6
Partner	Yes	12.2	12.5	15.3	15.6
	No	47.2	57.0	25.0	32.8
Domicile	Urban	17.2	31.4	17.3	23.3
	Rural	23.7	34.5	17.7	23.4
Health	Good	14.4	24.3	9.1	11.1
	Fair	23.3	35.3	22.5	27.0
	Poor	32.6	48.4	35.9	46.7
Education	Low	30.7	44.9	25.6	30.7
	Intermediate	17.0	25.4	15.2	19.1
	High	5.9	14.9	6.9	12.3
In paid work	Yes	8.8	14.7	9.9	14.6
	No	22.8	35.8	19.4	24.9
Regime	Continental	16.1	25.3	9.7	12.1
	Southern European	23.9	37.1	29.1	39.1
	Anglo-Saxon	28.3	32.2	9.5	10.9
	Eastern European	22.2	40.8	32.5	39.5
	Nordic	16.3	27.9	6.6	8.0

Percentages are calculated on weighted data (pweight and pspwght)

**Table 3:** Multilevel regression models of objective poverty on gender and control variables

	M0			M1			M2			M3			M4		
	OR		S.E	OR		S.E	OR		S.E	OR		S.E	OR		S.E
Constant	.398	***	.037	.444	***	.056	2.130	***	.322	.282	***	.015	2.083	***	.379
*Individual level:*															
Female gender				1.267	***	.064	1.161	**	.062				1.160	**	.062
Age 70 years or more				1.400	***	.069	.922		.050				.922		.050
Living with a partner				.118	***	.006	.113	***	.006				.113	***	.006
Rural residence				1.601	***	.081	1.340	***	.071				1.340	***	.071
Bad health (ref.)															
Fair health							.802	**	.059				.802	**	.059
Good health							.802	***	.059				.577	***	.045
Low education (ref.)															
Intermediate education							.424	***	.025				.421	***	.025
High education							.173	***	.016				.173	***	.016
Still working							.387	***	.034				.388	***	.034
*Country level:*															
Continental										.078		.051	.225		.180
Anglo-Saxon										.606		.618	.048		.149
Eastern European										.618		.370	.196		.203
Southern European										.235		.205	.080		.123
Level-2 variance (S.E)	.171		.056	.168		.056	.179		.060	< 0.01		< 0.01	.045		.048
Approx. VPC (%)	5.0			4.9			5.0			< 0.01			1.4		
N		10,900			10,954			10,954			10,960.00				
Log likelihood		−6468.765			−5263.802			−4860.98			−6465.352			−4859.432	
AIC		12,941.53			10,539.6			9743.96			12,942.7			9748.863	
BIC		12,956.13			10,583.41			9824.276			12,986.52			9858.385	

The table reports odds ratios, standard errors (S.E) and variance components of individuals nested in 21 countries. *p < 0.05, **p < 0.01, ***p < 0.001

**Table 4:** Multilevel regression models of subjective poverty on gender and control variables

	M0			M1			M2			M3			M4		
	OR		S.E	OR		S.E	OR		S.E	OR		S.E	OR		S.E
Constant	.206	***	.044	.297	***	.069	1.593	*	.355	.110	***	.017	.958		.185
*Individual level:*															
Female gender				1.090		.060	1.002		.569				1.001		.057
Age 70 years or more				.886	*	.047	.598	***	.035				.598	***	.035
Living with a partner				.393	***	.022	.424	***	.024				.425	***	.024
Rural residence				1.112	*	.060	.951		.054				.954		.054
Bad health (ref.)															
Fair health							.562	***	.040				.563	***	.040
Good health							.282	***	.023				.283	***	.023
Low education (ref.)															
Intermediate education							.511	***	.033				.511	***	.033
High education							.276	***	.028				.276	***	.028
Still working							.546	***	.049				.543	***	.049
*Country level:*															
Continental										< 0.01		< 0.01	< 0.01		.000
Anglo-Saxon										.002		.262	.119		.339
Eastern European										2.74		1.80	2.047		1.368
Southern European										2.20		2.05	1.254		1.243
Level-2 variance (S.E)	.934		.296	.920		.291	.718		.229	.212		.117	.179		.099
Approx. VPC (%)	22.1			21.8			17.9			6.0			5.2		
N		10,960			10,954			10,954			10,960			10,954	
Log likelihood		−4915.22			−4745.846			−4409.156			−4911.871			−4406.488	
AIC		9834.439			9503.692			8840.312			9835.741			8842.976	
BIC		9849.043			9547.501			8920.628			9879.553			8952.497	

The table reports odds ratios, standard errors (S.E) and variance components of individuals nested in 21 countries. * p < 0.05, ** p < 0.01, *** p < 0.001

## Findings

**Fig. 2:**
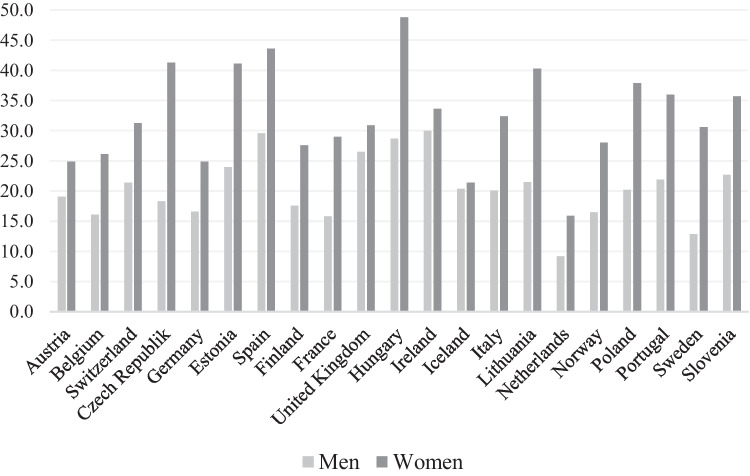
Objective poverty (%) in older men and women (60+), by country

**Fig. 3:**
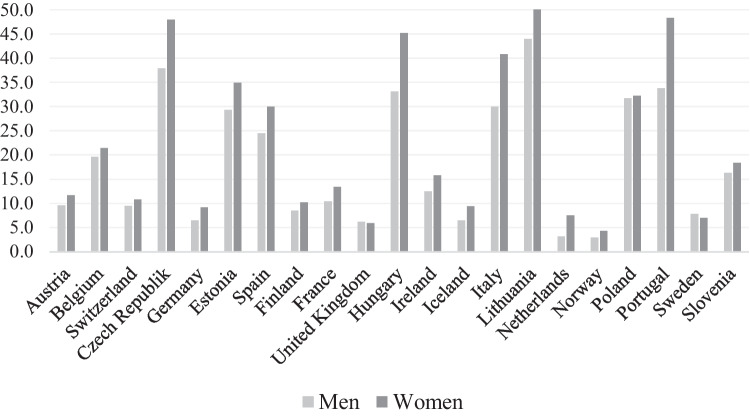
Subjective poverty (%) in older men and women (60+), by country

The results show that both objective and subjective poverty is more common in women than in men (Fig. 1). The objective poverty rate is 32.7 percent in women and 20.0 percent in men. The female and male subjective poverty rates are 23.4 and 17.5, respectively. Women have higher rates of objective poverty than men in each country studied (Table 2 and Fig. 2) and higher subjective poverty rates in all countries except Sweden and the United Kingdom (Fig. 3 and Table 2). Hence, all welfare regimes show higher objective and subjective poverty incidence in women than men. As to other control variables, objective poverty rates are higher among both men and women who are aged seventy or more as compared to the younger ones. In the oldest women, as an example, the objective poverty rate is 41.3, while it is 24.9 in the younger group of women (Table 2). Those living with no partner also show disproportionately high objective poverty rates. In women, the percentage is almost sixty and in men close to fifty. Respondents who live in rural areas, who have low education, who report poor or fair health and who are not in paid work are also over-represented when poverty is measured objectively. The overall pattern for subjective poverty is similar.

Overall, objective poverty is more common than subjective poverty, but the latter is more prevalent in both gender in the Czech Republic, Italy, Lithuania and Portugal, and in men particularly in Estonia, Hungary and Poland, but also in Belgium.

The results of the multilevel logistic regression analyses are presented in Table 3 (objective poverty) and Table 4 (subjective poverty). As to objective poverty, we see that female gender remain positively and significantly associated with objective poverty throughout the models. However, we also see that this association weakens when controlling for other individual-level variables (M1 and M2 in Table 3) whereas the association with welfare regimes remain insignificant (M3 and M4 in Table 3). Adjusted for all control variables, the odds ratio of being found in objective poverty is 16 percent higher for women than men (M4 in Table 3). To live with a partner, to work, to have intermediate or high education and to have good or fair subjective health significantly decreases the odds of objective poverty, while being a rural resident significantly increases the odds of being found in this kind of poverty. In model 1, persons being 70 years or older are more likely than the younger groups to be found in objective poverty, but this difference turns insignificant when controlling for other variables (M2–M4). Continental, Anglo-Saxon, Eastern and Southern welfare regimes are all more positively correlated with objective poverty than the Nordic, and this particularly relates to the Anglo-Saxon and Eastern regimes, although the estimates are not significant (M3 and M4 in Table 3).

Interestingly, we find no significant gender differences in subjective poverty (Table 4) when simultaneously controlling for other variables. Neither are there any significant differences in this kind of poverty between urban and rural residents. Reporting good or fair health, having high or intermediate education, living with a partner, being in work and being 70 years or older are all characteristics that significantly decreases the odds for subjective poverty. As to welfare regimes, we note a similar pattern as for objective poverty, since all regimes are more positively associated with subjective poverty than the Nordic regime, and this is especially true for the Eastern and Southern regimes. But also here the estimates remain insignificant.

The baseline model (M0) in Table 3 shows that 5.0 percent of the total variance of objective poverty resides on the contextual level, while a notably larger share, 22.1 percent, of the variance in subjective poverty resides on a contextual level (M0 in Table 4). In the full models in each table, which take all control variables into account, the percentages are lower, or 1.4 in objective poverty and 5.2 in subjective poverty. This would suggest that the variance in objective poverty stems from factors at the individual level rather than contextual factors.

## Discussion

This paper has been concerned with gendered old-age poverty in 21 European countries, with the overall aim to study objective and subjective income poverty among older females and males and the role that gender and other individual-level variables as well as contextual factors plays herein. It has examined the prevalence of objective and subjective poverty among older women and men, analyzed whether this prevalence is explained by gender while simultaneously controlling for other individual-level variables and studied the role of context by assessing how much of the poverty variance resides on a country level and what the explanative power of different welfare state arrangements is.

Based on our findings, a number of conclusions can be drawn. First, this paper shows that older females evidently face higher risks of being income-poor than older males do. This applies to objective poverty in all countries studied, and to subjective poverty in a vast majority of the countries. After controlling for factors at the individual level as well as for the welfare regimes, the gender differences in objective poverty remain. However, there is a clear association between objective poverty and the control variables used, suggesting that this kind of poverty has a tendency to be higher among persons living in rural areas and lower among those living with a partner, those reporting good health, better-educated persons and among elderly who are in paid work. Although no significant correlation between objective poverty and welfare regimes are found, the estimates indicate that old-age objective poverty is more common in Continental, Anglo-Saxon, Eastern and Southern welfare state arrangements than in Nordic countries.

In contrast to objective poverty, gender differences in subjective poverty are found to be negligible. However, the pattern in the association between subjective poverty and the control variables is similar to the one for the relationship between objective poverty and the control variables. As an exception, though, subjective poverty is not associated with living in a rural residence, suggesting that among older persons, those in rural areas in general are more probable to feel satisfied with their economic situation as compared to their counterparts in urban areas. Finally, we note that subjective poverty is more prevalent than objective amongst men in the countries categorized as Eastern European regimes except for Slovenia, as well as in women in the Czech Republic and Poland, and in both gender in Italy and Portugal. We can only speculate on the reasons, which might be related to national income per capita as well as the previously relatively low possibilities to gain wealth in post-communist countries which is reflected in difficulties to make ends meet in old age. Traditional male wage earner norms in some of these countries may also play a role.

To sum up the findings so far, objective old-age poverty seems to be more prominent from a gender perspective, while subjective old-age poverty appears more closely related to socioeconomic factors, health and living conditions. A tentative explanation of this result could be that women in these cohorts have had lower incomes than men during their working lives, and that they have adapted to this situation by fostering lower expectations as to their income situation when becoming retired. The fact that those aged 70 years or older experience lower risks of subjective poverty may also imply the interference of age cohort effects suggesting that those born in the 1940s may have lower income expectations than those born in the 1950s. However, it should be noted that both objective and subjective poverty is strongly related to partnership and whether one is still doing paid work as well as health and education. To pool incomes (and wealth) with a partner does not only lower poverty risks objectively speaking, but may also influence income expectations and subjective experiences of poverty.

To some extent these findings are in accordance with those found in previous comparative research on old-age poverty, such as Smeeding and Sandström (2005), Ahonen and Bach-Othman (2010) and Möhring (2015), who have found a higher prevalence of poverty among women than men. These studies have also shown that the incidence of poverty tends to increase with higher age and changing living situations from partnership to living alone, but to be negatively associated with better health and higher education. The present results also corroborate findings from earlier old-age poverty studies that found smaller gender differences in subjective poverty than objective (Härtull and Nygård 2014; Nygård et al. 2017). However, the insignificant gender differences in subjective poverty, when examining the importance of control variables that are reported in the current paper do not fully support the suggestion that female poverty may be underestimated if subjective perceptions of poverty are neglected (Kabeer 2015; Kwan and Walsh 2018; Lister 2004).

Another conclusion that arises from the results relates to the proportion of poverty variance that originates from the contextual level. When gender and all control variables are included in the objective and subjective models, the VPCs are 1.4 and 5.2, respectively. Thus, not only is objective poverty more prevalent than subjective, but most of the variance in it stems from differences within countries. The present study does not point to any significant explanative power of different welfare state arrangements as such, but pension systems, living arrangements among elderly and other structural factors within countries might nevertheless play a role.

A somewhat higher share of the variance in subjective poverty originates from the contextual level than the variance in objective poverty, suggesting that subjective experiences of poverty are more influenced by factors such as price levels, pensions and sociocultural norms that may influence the ways in which older people view their economic situation across countries.

One limitation of this study relates to the method used in the analysis. Since the number of countries in the multilevel regressions is relatively small, the estimates and standard errors might be biased (see e.g., Bryan and Jenkins 2016). Another limitation relates to the way that incomes are surveyed in ESS. It is possible that older persons could face difficulties in estimating in which income decile their household’s net income would fit, which in turn may affect the results as regards objective poverty. Our study is also limited by the lack of information on wealth, because material and economic resources, which we are not able to control for, are essential factors in subjective experiences of poverty (Brulé and Suter 2019; Headey et al. 2008). Finally, measuring the role of welfare state configurations on the contextual level with the help of crude dummies based on country clusters can be seen as problematic, since old-age poverty varies within welfare regimes and there are differences in terms of pension rights and replacement rates, even within welfare regimes. We therefore suggest that further research analyze contextual-level welfare state characteristics with the help of more nuanced variables.

Gendered poverty does certainly seem to be a problem facing today’s older populations in Europe, in particular when it comes to poverty based on an objective measure. Although the subjective experience must not be neglected, this is something that needs to be addressed and discussed in the public sphere, not only because it may have implications for the overall well-being of older women, but also because of the rising age of populations in this part of the world, a population that is expected to be predominately female.

## Footnotes

1 The present study was started before the COVID-19 pandemic but delayed due to pandemic-related issues. Hence, the use of the 2016 round of the European Social Survey.

2 We designed a new weight variable based on the post-stratification and population size weights, and used the *svyset* command to modify the analyses according to a survey data structure.
